# 975 nm Diode Laser-Mediated Dentin Surface Modification Enhances Bio-ceramic Sealer Retention: Comparative Study with Er: YAG Photoacoustic Irrigation

**DOI:** 10.12669/pjms.42.8.16385

**Published:** 2026-08

**Authors:** Amer M. Alanazi, Azmat Ali Khan

**Affiliations:** 1 Amer M. Alanazi, Pharmaceutical Biotechnology Laboratory, Department of Pharmaceutical Chemistry, College of Pharmacy, King Saud University, Riyadh 11451, Saudi Arabia; 2 Azmat Ali Khan, Pharmaceutical Biotechnology Laboratory, Department of Pharmaceutical Chemistry, College of Pharmacy, King Saud University, Riyadh 11451, Saudi Arabia

**Keywords:** Bioceramic sealers, Diode laser, Photon-Induced Photoacoustic Streaming, Sodium hypochlorite

## Abstract

**Objectives:**

To evaluate different irrigation protocols, Photon-Induced Photoacoustic Streaming (PIPs) using Sodium hypochlorite (NaOCl) and NaOCl with EDTA and diode laser (DL) compared to syringe irrigated (SI)-NaOCl+EDTA (control) on smear layer (SL) and dislocation resistance of Bioceramic (BC) sealers to canal dentin.

**Methodology:**

This in vitro experimental study was approved by the institutional review board, King Saud University CDRC# FC-58-41-2026. The duration of the study was three months, January 1, 2026 - March 1, 2026. Seventy five central incisors were included. The prepared specimens were randomly divided into three experimental groups according to the disinfection protocol. *Group-1:* SI-NaOCl+EDTA (Control), *Group-2:* PIPS-NaOCl+EDTA, and *Group-3:* NaOCl+ EDTA+DL. On five specimens from each category, SL removal was assessed using a scanning electron microscope (SEM). Ten specimens from each category were obturated with premixed Endosequence BC sealer (EBCS), and another 10 were bonded with two-component BioRoot RCS (BRS). The Universal Testing Machine was used to assess dislocation resistance. The failure type was evaluated using a stereomicroscope. ANOVA with post hoc comparisons of SL elimination and dislocation resistance among the tested categories.

**Results:**

Group-3 NaOCl+EDTA+DL specimens displayed the highest SL removal and maximum scores of dislocation resistance. Group-1 SI-NaOCl+EDTA-treated canals showed the lowest SL removal and the lowest dislocation resistance.

**Conclusion:**

DL as an adjunctive therapy after conventional syringe irrigation utilizing NaOCl and EDTA displayed the most satisfactory performance in terms of removing SL and improving the dislocation resistance of different bioceramic sealers.

## INTRODUCTION

Endodontic sealers play a critical role in reducing infections and ensuring the success of root canal therapy. Effective adherence of these sealers to canal dentin provides resistance to microleakage and entraps residual microorganisms within the complex anatomy and dentinal tubules.^1^ However, achieving optimal sealer penetration requires thorough removal of the smear layer (SL) lining canal walls, as this debris prevents tubular penetration and compromises sealer retention to root canal dentin.^2^

Sodium hypochlorite (NaOCl) and ethylenediamine tetraacetic acid (EDTA) are used in combination for root canal disinfection and smear layer removal. NaOCl is the primary irrigant due to its low viscosity, broad-spectrum antimicrobial activity, and ability to dissolve organic tissue.^3^ EDTA, as a chelating agent, eliminates inorganic components of the smear layer and exposes dentinal tubules. This NaOCl-EDTA protocol represents the standard approach for root canal cleaning and disinfection.^4^ These irrigants are delivered via manual syringe irrigation (SI) with needles of various gauges and vent designs.^5^ However, this method is limited by the complexity of the root canal system and syringe-needle delivery constraints, necessitating more effective alternatives.

Dental lasers offer promising approaches for root canal debridement and disinfection through laser-activated irrigation (LAI) techniques.^6^ LAI induces cavitation and acoustic streaming in intracanal fluids via photomechanical laser energy at reduced power settings. Erbium lasers exhibit effective absorption in water and NaOCl, inducing vaporization and bubble formation that enhance irrigation efficiency in anatomically challenging regions such as the apical third.^7^ Lasers can also function as adjunctive treatments following conventional NaOCl and EDTA irrigation.^8^ Laboratory studies by Amin et al. showed that diode laser irradiation effectively enhances smear layer removal and exhibits bactericidal activity against endodontic pathogens.^9^ However, its efficacy as an adjunct for smear layer removal and its influence on root canal sealer retention require further evaluation.

Bioceramic (BC) sealers are an emerging class of endodontic materials that establish hermetic seals between root canal dentin and filling materials. These hydraulic cements set upon exposure to moisture, releasing calcium ions while maintaining alkaline pH that promotes hydroxyapatite formation, conferring bioactive properties.^10^ EndoSequence BC sealer (EBCS) is a premixed, aluminum-free formulation. Recent work by Srivastava et al. reported that EBCS has inferior bond strength compared with AH Plus resin-based sealer.^11^ BioRoot RCS (BRS) is a two-component system comprising a powder phase (tricalcium silicate, zirconium oxide, povidone) and a liquid phase (calcium chloride, polycarboxylate). BRS shows superior sealing in hydrophilic environments through mineralization at the root canal interface.^12^ Findings by Yaduka et al. indicated that BioRoot RCS exhibited greater resistance to dislocation than AH Plus.^13^ Despite these advances, definitive recommendations for selecting the optimal bioceramic sealer after laser-assisted irrigation protocols remain unavailable.

The present investigation was designed to test two null hypotheses: (1) no statistically significant differences would exist in smear layer removal effectiveness among root canals treated with photon-induced photoacoustic streaming (PIPs) using NaOCl + EDTA, NaOCl + EDTA + diode laser, compared to conventional syringe irrigation with NaOCl + EDTA; and (2) comparable dislocation resistance would be observed between root canals obturated with premixed EBCS versus two-component BRS when identical irrigation protocols were employed. Therefore, this study aimed to evaluate the influence of various irrigation techniques on smear layer removal and the displacement resistance of different bioceramic sealers to root canal dentin when identical irrigation protocols are employed.

## METHODOLOGY

This in vitro experimental study adhered to ethical guidelines for research involving extracted human teeth, with approval obtained from the institutional review board CDRC# FC-58-41-2026. The duration of the study was three months, 1^st^ January 2026 - 1^st^ March 2026.

### Sample selection, Preparation, and Sample size:

### calculation:

This existing inquiry involved 75 human central incisors with fully formed roots. Inclusion criteria followed teeth with single, straight root canals confirmed via periapical radiographs (buccolingual and mesiodistal projections) and absence of root resorption. By using the WHO sample size calculator, confidence level = 95%, absolute precision = 9%, population mean = 11.40, population standard deviation = 0.19, a sample size of = 25 cases in each group was estimated.^14^,^15^

### Specimen processing:

Roots were mechanically debrided using a periodontal curette. Crowns were removed perpendicular to the long axis using a water-cooled slow-speed diamond fissure bur (#016, Komet, Rock Hill, SC, USA), establishing a standardized 12 mm root length measured from the apex using a digital caliper.^16^

### Endodontic preparation:

Root canal access was established, and canal patency was confirmed using a #10 K-file (Dentsply Maillefer, Ballaigues, Switzerland). Working length was determined 1 mm short of the apical foramen using an apex locator (Root ZX mini, J. Morita, Tokyo, Japan), verified radiographically. Canal shaping was performed using the ProTaper Universal rotary system (Dentsply Maillefer) in a crown-down sequence at 300 rpm using an endodontic motor (X-Smart, Dentsply Maillefer).^15^

### Final Irrigation Protocol Groups:

Specimens were randomly allocated into three groups (n=25 per group) using a computer-generated simple random sampling method.

### Group-1:

Conventional Syringe Irrigation (SI-NaOCl + EDTA). 5 mL of 2.5% sodium hypochlorite was delivered via a 30-gauge side-vented needle for 30 seconds with gentle agitation (2 mm vertical amplitude**)**. 3 mL of 17% EDTA solution (Colgate Oral Care, Waverly, Australia) was delivered for 60 seconds using an identical technique 2 mm short of working length throughout.^16^

### Group-2: Photon-Induced Photoacoustic Streaming (PIPS-NaOCl+EDTA):

3 mL of 2.5% sodium hypochlorite activated using Er: YAG laser (KEY Laser, KaVo, Biberach, Germany; wavelength: 2940 nm) in PIPS mode with the following laser parameters: Pulse energy: 20 mJ; Frequency: 15 Hz; Pulse duration: 50 μs; and fluence: 2.06 J/cm². A radial PIPS tip (600 μm diameter, 9 mm length) positioned in the pulp chamber without canal contact, with 30 seconds of continuous activation, solution delivered via a stationary needle. The residual NaOCl was aspirated using a syringe. This was followed by 3 mL of 17% EDTA activated for 60 seconds total (three 20-second cycles with solution replenishment between cycles) with complete removal of residual irrigant between solutions.^6^

### Group-3: Diode Laser Adjunctive Treatment (NaOCl + EDTA + DL):

Initial irrigation identical to Group-1 (NaOCl + EDTA protocol) followed by Diode laser irradiation (Sirona, Lite Medics, Italy): with the following parameters: Wavelength: 975 nm; Power: 2 W (continuous wave); Pulse mode: 5 ms ON, 25 ms OFF, and Optical fiber: 320 μm diameter, 0.22 numerical aperture. The irradiation protocol was 60 seconds divided into three 20-second intervals with 10-second rest periods. A diode laser fiber was inserted 1 mm coronal to the working length with continuous helical motion (apical-coronal) at approximately 2 mm/second to ensure uniform energy distribution and prevent thermal accumulation.^6^,^16^

### Scanning Electron Microscopy (SEM) Analysis- Sample Preparation and Assessment:

Five specimens per group (n=5 total) were selected using simple random sampling. The roots were sliced longitudinally. Each segment was sequentially dehydrated in a graded ethanol series, mounted on aluminum stubs, and sputter-coated with a gold-palladium alloy. Each segment was examined using SEM at 20 kV and at different magnifications to assess smear layer removal. SL removal was scored independently by two calibrated examiners (inter-examiner reliability: κ=0.89) using the Hülsmann scoring system.^17^

### Root Canal Obturation and Sample Sectioning:

Remaining specimens (n=20) were subdivided based on Bioceramic sealer type (n=10 per subgroup). Subgroup A: EndoSequence BC Sealer (EBCS). Premixed bioceramic paste (Brasseler USA, Savannah, GA, USA). Subgroup B: BioRoot RCS (BRS)A two-component bioceramic sealer (Septodont, Saint-Maur-des-Fossés, France). ***Synthetic Aging:*** All segments were thermocycled (Automatic Thermocycling Dipping Machine) to simulate temperature fluctuations in the mouth. The specimens underwent 10,000 cycles using two water baths. One functioned continuously at 5°C while the other functioned at 55°C.^14^

### Assessment of Dislocation Resistance:

Universal testing machine (Hounsfield Test Equipment, Redhill, UK) equipped with custom stainless steel cylindrical plungers (diameters: 0.4 mm, 0.7 mm, 0.9 mm selected based on canal diameter at each level. Specimens were positioned on a custom jig with a central hole allowing unobstructed apical displacement. The plunger was aligned perpendicular to the slice surface.

### Failure mode analysis:

All debonded specimens were examined under stereomicroscopy (M165 FC, Leica Microsystems, Wetzlar, Germany) at ×40 magnification by two calibrated observers (inter-rater reliability: κ=0.92). Failure types were classified as adhesive, cohesive, and admixed failure.

### Statistical analysis:

Data were analyzed using SPSS version 26.0 (IBM Corp., Armonk, NY, USA). Normality was assessed using the Shapiro-Wilk test. Two-way ANOVA was used to evaluate differences in SL scores and the dislocation assessment among groups. Post Hoc Tukey’s test was used to determine notable differences among groups in SL removal and assessment of dislocation (p = 0.05). Failure mode was given in percentages.

## RESULTS

### SL removal Assessment:

**Fig.1:**
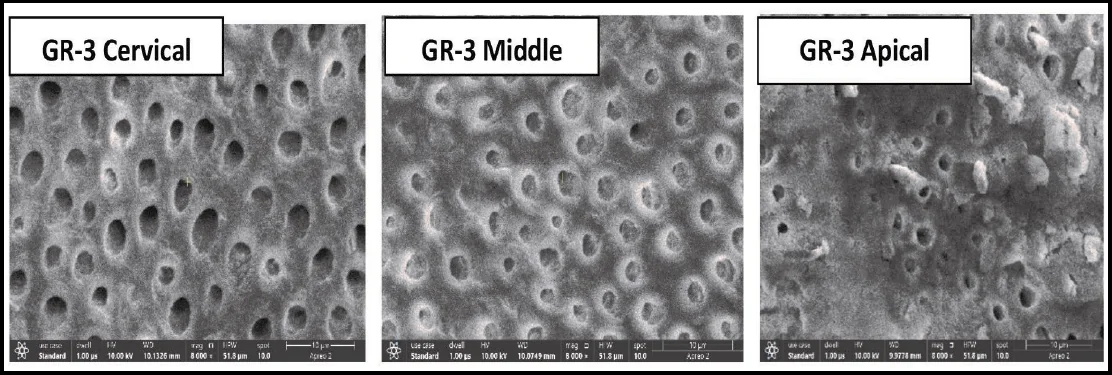
SEM images of coronal, middle, and apical regions when the sample was irrigated with NaOCl + EDTA + DL.

In Table-I, the SL removal from the root canal after using different disinfection protocols. The cervical section of Group-3 specimens displayed the highest SL removal. Whereas, the apical section of Group-1 (SI-NaOCl + EDTA) treated canals presented the lowest SL removal. Intergroup comparison analysis showed that all three groups, i.e., Group-1, Group-2 (PIPS-NaOCl + EDTA), and Group-3 (NaOCl+EDTA+DL), displayed significantly different outcomes of SL elimination in all three coronal, middle, and apical (p<0.05) (Fig.1).

**Table-I:** Smear layer removal from the canal employing different disinfection protocols.

Tested categories	Mean ± SD Cervical	Mean ± SD Middle	Mean ± SD Apical	p-value!
Group-1: SI-NaOCl + EDTA	3.98±0.18 ^c,A^	4.21±0.12 ^c,A^	4.82±0.43 ^c,B^	p<0.05
Group-2: PIPS-NaOCl + EDTA	2.54±0.22 ^b,A^	2.76±0.21 ^b,A^	3.55±0.13 ^b,B^	
Group-3: NaOCl + EDTA + DL	1.75±0.37 ^a,A^	1.87±0.42 ^a,A^	2.53±0.23 ^a,A^	

Sodium hypochlorite (NaOCl), Ethylenediamine tetraacetic acid (EDTA), Photon-Induced Photoacoustic Streaming (PIPS), Diode laser (DL). ! ANOVA Different superscript lower-case alphabets denote statistically significant differences within the same column (p < 0.05). Data with different upper-case letters denote significant differences within each row (p < 0.05), Post Hoc Tukey.

### Dislocation resistance assessment:

The dislocation resistance of different bioceramic sealers bonded to root canal dentin after employing different canal disinfectants. The cervical part of the 3A specimens showed the highest dislocation resistance scores. Whereas, the minimum dislocation resistance was observed in the apical part of Group-1B. Comparative analysis among the investigated categories showed that Group-2A (PIPS-NaOCl + EDTA –EBCS) and Group-2B (PIPS-NaOCl + EDTA-BRS) exhibited similar dislocation resistance, with no statistically significant difference (p>0.05). Likewise, Group-1A (SI-NaOCl+EDTA-EBCS) and Group-1B (SI-NaOCl+EDTA-BRS) also showed comparable bond integrity(p>0.05) in all three thirds. Similarly, Group-3A (NaOCl + EDTA + DL-EBCS) and Group-3B (NaOCl + EDTA + DL-BRS) also showed comparable dislocation resistance values (p>0.05) for the cervical, middle, and apical regions, shows in Table-II.

**Table-II:** Dislocation resistance of bio-ceramic sealers bonded to root canal dentin after employing different disinfectant regimes.

Tested categories	Mean ± SD Cervical	Mean ± SD Middle	Mean ± SD Apical	p-value!
Category 1A: SI-NaOCl + EDTA-EBCS	7.63±0.12 ^c,A^	7.35±0.21 ^c,A^	6.24±0.32 ^c,B^	p<0.05
Category 1B: SI-NaOCl + EDTA-BRS	7.33±0.15 ^c,A^	7.18±0.11 ^c,A^	6.12±0.41 ^c,B^	
Category 2A: PIPS-NaOCl + EDTA -EBCS	10.22±0.27 ^b,A^	9.95±0.18 ^b,A^	8.78±0.17 ^b,B^	
Category 2B: PIPS-NaOCl + EDTA-BRS	10.01±0.36 ^b,A^	9.84±0.14 ^b,A^	8.61±0.12 ^b,B^	
Category 3A: NaOCl + EDTA + DL-EBCS	11.53±0.43 ^a,A^	11.24±0.33 ^a,A^	11.12±0.23 ^a,A^	
Category 3B: NaOCl + EDTA + DL-BRS	11.43±0.34 ^a,A^	11.17±0.21 ^a,A^	11.01±0.12 ^a,A^	

Sodium hypochlorite (NaOCl), Ethylenediamine tetraacetic acid (EDTA), Photon-Induced Photoacoustic Streaming (PIPS), Diode laser (DL) ! ANOVA Different superscript lower-case alphabets denote statistically significant differences within the same column (p < 0.05). Data with different upper-case letters denote significant differences within each row (p < 0.05), Post Hoc Tukey.

### Failure mode assessment:

**Fig.2:**
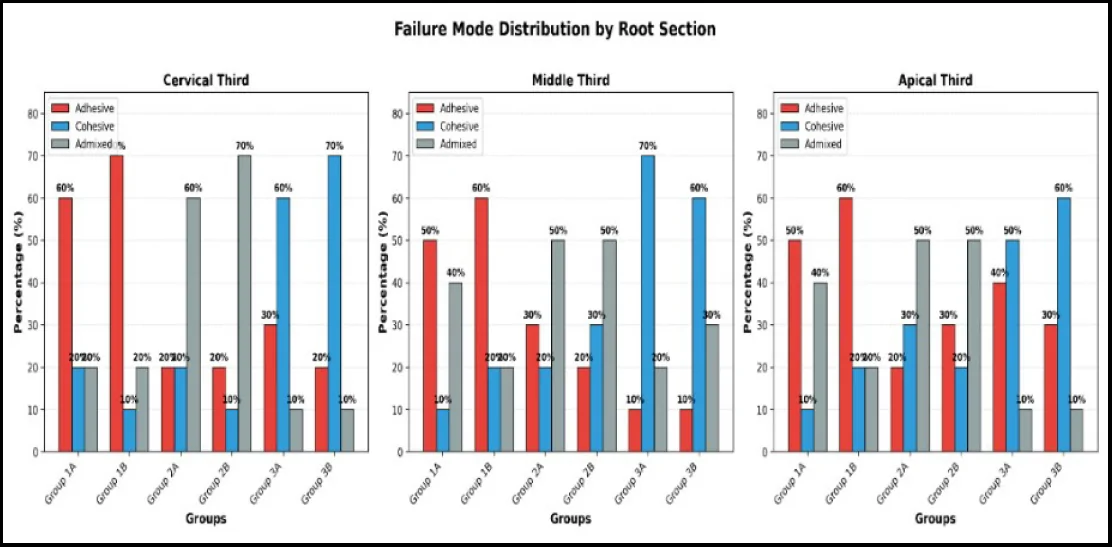
Grouped bar chart showing the type of failure by root section.

In Fig.2, illustrates the distribution of failure modes across cervical, middle, and apical root sections following different irrigation protocols. Groups-1A and 1B (conventional syringe irrigation) demonstrated predominantly adhesive failure. Groups-2A and 2B (PIPS-activated irrigation) exhibited primarily admixed failure patterns (50-70%). In contrast, Groups-3A and 3B (diode laser adjunctive treatment) showed cohesive failure dominance across all root sections.

## DISCUSSION

The present investigation evaluated two null hypotheses regarding endodontic irrigation protocols and bioceramic sealer performance. The first hypothesis posited that PIPS with NaOCl + EDTA and conventional NaOCl+EDTA with DL would demonstrate equivalent smear layer removal efficacy and dislocation resistance compared to conventional SI alone. The second hypothesis predicted comparable dislocation resistance between premixed EBCS and two-component BRS when subjected to identical irrigation protocols. Based on the experimental findings, the first null hypothesis was comprehensively rejected. Conversely, the second hypothesis was accepted, as both bioceramic sealers demonstrated statistically comparable dislocation resistance when identical disinfection protocols were employed.

Root canals treated with NaOCl + EDTA + DL showed superior dislocation resistance compared to PIPS-activated and conventional syringe irrigation protocols across all root thirds. These findings corroborate ex vivo investigations by Bago et al., who reported maximum bond strength values following diode laser irradiation as an adjunct to conventional irrigation throughout cervical, middle, and apical canal sections.^18^ The enhanced performance results from the photothermal effects of 975 nm diode laser irradiation on root canal dentin, which produce ultrastructural modifications.^18^ Naghsh et al. documented that laser energy at 810-980 nm induces microstructural alterations in intertubular dentin, increasing surface roughness and creating retention sites that facilitate better sealer adaptation. Photothermal activation opens dentinal tubule orifices while maintaining dentin microhardness, thereby establishing an optimal substrate for sealer infiltration.^19^

Specimens treated with PIPS showed intermediate dislocation resistance, with superior performance versus conventional SI but lower values than diode laser-supplemented irrigation. The PIPS mechanism uses Er: YAG laser energy (2940 nm) to generate vapor bubble formation, collapse, and acoustic streaming within intracanal irrigants. This photomechanical effect enhances fluid circulation throughout the root canal, improving debris and SL removal compared with passive irrigation.^20^ Recent studies by Akcay et al. confirmed that PIPS activation using EDTA (30 seconds) followed by NaOCl (30 seconds) enhanced epoxy resin-based sealer bond strength versus conventional irrigation.^21^ Wen et al. showed that PIPS produces stronger effects in coronal regions, with increased dentinal tubule exposure in cervical sections. At the same time, residual smear layer and debris persisted more in apical dentin walls.^6^ This variation has been attributed to attenuation of acoustic wave intensity apically and challenges with irrigant exchange in confined apical anatomy.^6^ Seghayer et al. suggested that reduced PIPS effectiveness in apical thirds stems from decreased shock wave propagation combined with higher debris accumulation away from the laser tip.^7^

Conventional syringe irrigation showed the lowest smear layer removal scores and dislocation resistance among the tested protocols. Despite widespread clinical adoption, SI has fundamental limitations in achieving irrigant penetration beyond the needle tip, particularly in apical regions where anatomical constraints restrict fluid circulation.^5^ Positive-pressure irrigation with side-vented needles positioned 2 mm short of the working length fails to displace apical air, limiting irrigant exchange in terminal canal segments. This “vapor lock effect” prevents adequate contact between NaOCl and EDTA at apical dentin surfaces, resulting in persistent smear layer coverage that impedes sealer adaptation and tubular penetration.^4^ Consequently, the sealer-dentin interface in SI-treated specimens remains compromised by residual debris, which explains the reduced dislocation resistance and the predominant adhesive failure patterns in this group.

Comparative analysis of the two bioceramic formulations revealed that premixed EBCS exhibited marginally superior dislocation resistance compared with two-component BRS; however, this difference was not statistically significant (p>0.05). These findings contrast with previous laboratory investigations by Donnermeyer et al., who reported that monophasic calcium silicate-based sealers had significantly higher push-out bond strength than biphasic formulations.^22^ Conversely, studies by Falakaloğlu et al. documented superior performance of BRS relative to other premixed bioceramic alternatives, indicating variability in comparative sealer performance across different experimental conditions and testing protocols.^23^ The modest numerical advantage observed for EBCS may be attributed to its premixed formulation, which ensures consistent material composition, eliminates mixing variables, and provides immediate, ready-to-use application.^24^ This manufacturing approach guarantees uniform particle distribution, optimal powder-to-liquid ratios, and standardized rheological properties that facilitate predictable sealer flow and dentin adaptation.^25^

### Study Limitations:

Several methodological constraints warrant acknowledgment when interpreting these findings. As an in vitro investigation, the push-out test provides a controlled assessment of interfacial mechanics but cannot fully replicate the complex biomechanical environment encountered by root canal fillings in vivo. The present investigation used a single standardized parameter for both laser irradiation (975 nm wavelength, 2 W power, specific pulse configuration) and chemical irrigant concentrations (2.5% NaOCl, 17% EDTA).

### Study Novelty and addition to existing Dental Literature:

This study is among the first to directly compare 975 nm diode laser adjunctive irradiation with photon-induced photoacoustic streaming (PIPS) using an Er: YAG laser, specifically in the context of bioceramic sealer dislocation resistance to root canal dentin. This outcome has received limited attention relative to conventional resin-based sealers. A key distinguishing feature is the simultaneous evaluation of two chemically distinct bioceramic systems under identical irrigation conditions, enabling direct comparison of sealer-specific responses to laser-modified dentin surfaces within a single standardized experimental model. The current findings challenge the prevailing clinical preference for photoacoustic activation by demonstrating that a simpler, more widely accessible adjunctive laser modality can achieve equivalent or superior outcomes, thereby offering a more practical and cost-effective alternative for clinicians without access to Er: YAG laser systems.

## CONCLUSION

Diode laser irradiation, used as an adjunct to conventional syringe irrigation with sodium hypochlorite and ethylenediamine tetraacetic acid, showed superior smear layer removal and enhanced resistance to dislocation of bioceramic root canal sealers. Both premixed and two-component bioceramic sealer formulations exhibited comparable retention under equivalent irrigation protocols.

### Future direction:

Future investigations should incorporate dose-response analyses to establish evidence-based clinical protocols tailored to diverse anatomical presentations and clinical scenarios.

### Authors’ Contribution:

**AA and AAK:** Conceptualization Methodology, Resources & Validation.

**AA:** Software data curation and Formal analysis.

**AAK and AA;** Investigation original draft preparation writing and is responsible for the authenticity of the data and manuscript.
